# Ultra-High Response Detection of Alcohols Based on CdS/MoS_2_ Composite

**DOI:** 10.1186/s11671-021-03647-3

**Published:** 2022-01-06

**Authors:** Lei Liu, Weiye Yang, Hui Zhang, Xueqian Yan, Yingkai Liu

**Affiliations:** 1 Yunnan Key Laboratory of Opto-Electronic Information Technology, Yunnan Normal University, Kunming, 650500 China; 2 Institute of Physics and Electronic Information, Yunnan Normal University, Kunming, 650500 China; 3 Key Laboratory of Advanced Technique and Preparation for Renewable Energy Materials, Ministry of Education, Yunnan Normal University, Kunming, 650500 China

**Keywords:** Cadmium sulfide, Molybdenum disulfide, Biomimetic framework, Alcohols detection, Gas sensor

## Abstract

Hybrid CdS/MoS_2_ with branch and leaf shaped structures are successfully synthesized by hydrothermal method. It is applied to detect volatile organic compounds, a basic source of indoor air pollution with deleterious effects on the human health. The sensor based on CdS/MoS_2_ displays an outstanding response to alcohols among numerous gases. Their response to 100 ppm ethanol and isopropanol reaches 56 and 94, respectively. Benefiting from the dendrite-like biomimetic structure and synergy effect of CdS and MoS_2_, the sensor exhibits higher response than traditional gas sensor. For multiple alcohols, the limit of detection reached ppb level. In addition, by comparing the response of ethanol, isopropanol, isoamyl alcohol and their mixtures with acetone and methanal, a strong resistance interference is observed. This work proved that the modified detector holds broad promise in the detection of alcohols.

**Supplementary Information:**

The online version contains supplementary material available at 10.1186/s11671-021-03647-3.

## Introduction

Toxic volatile organic compounds (VOCs) have become global problems that endanger the deterioration of environmental pollution and directly affect human life [1, 2]. Studies have shown that prolonged exposure to VOCs can lead to adverse health issues such as nausea [3], headache [4] and mucosal irritation [5]. Alcohols as important components of VOCs are widely used in medical diagnosis, food industry, winemaking industry and modern bio-technologies [6]. Therefore, it is essential to monitor them in daily production life. Among various types of gas sensors, nanomaterials-based semiconductors have been considered suitable for gas sensing applications due to their abundant surface-active sites, low cost, small size and high surface reactivity [7], and charge disturbance on the surface is easily reflected in the transport characteristics [8, 9]. Recently, many nanostructured gas sensors with biomimetic framework have been reported and exhibited significantly improved characteristics [10, 11]. In particular, chalcogenides with nanostructures have attracted considerable attention, among which cadmium sulfide (CdS), with a band gap of 2.4 eV, is a typical n-type semiconductor material widely used in solar cells [12], photocatalysis [13], photoelectric devices [14] and gas sensors [15]. Zhu et al. [16] prepared an ethanol sensor of single crystal CdS nanowires and obtained response value of 14.9. Similarly, Fu et al. [17] demonstrated a VOCs sensor based on leaf-like CdS with response of 63–100 ppm isopropanol. The above-mentioned literatures revealed that CdS has great potential for gas sensing. Meanwhile, 2D layered molybdenum disulfide (MoS_2_) as another sulfide owns various applications [18, 19] due to its tunable band gap (1.2–1.9 eV) dependent on the number of layers and has dominated research hotpots. It is supposed that sensing mechanisms for organic vapors in 2D family materials are related to the change in conductivity and charge transfer process due to the different depletion layer and space layers [20–22]. For instance, Yan et al. [23] successfully synthesized SnO_2_@MoS_2_ composites and obtained a better ethanol response than pure SnO_2_. We investigated and found that MoS_2_ can be synthetically well incorporated into conventional CdS branches, which further enhance its sensing performance.

In this work, a simple hydrothermal method is used to synthesize the CdS/MoS_2_ branches and leaves (BLs). Among them, CdS functioned as branches for efficient transportation, and thin layer MoS_2_ leaves structure is obtained by increasing Mo source. Multiple analytical methods are used to further explore the morphology, microstructure, elemental composition and the valence state of materials. Based on the CdS/MoS_2_ BLs, we have fabricated sensors for detecting multiple VOCs and found that it represents a high and special response for detecting alcohols, including ethanol, propanol, iso-propanol, butanol, iso-butanol and iso-amyl alcohol. Compared with pure branch shaped CdS, the CdS/MoS_2_ composites achieve stronger gas sensing performance. Multiple repetitions over a month gave nearly same response. In the mixed solution of alcohols with methanal and acetone, it exhibited excellent selectivity to alcohols with strong anti-interference ability.

## Experimental Section

### Synthesis of CdS/MoS_2_ BLs

The CdS/MoS_2_ composites were synthesized via a hydrothermal process based on Zhang’s work [24]. In brief, 10 mmol of Cd(NO_3_)_2_·4H_2_O, 1 mmol of Na_2_MoO_4_·2H_2_O, and 30 mmol of CH_4_N_2_S were mixed and dissolved in 70 ml of deionized water under stirring at room temperature. Then, the mixture is put into a 100 mL Teflon-lined stainless-steel autoclave and maintained at 220 °C in an electric oven for 24 h. Meanwhile, the pure branch shaped CdS sample is synthesized in the same proportion without adding Mo source. After the reaction, it naturally cooled to room temperature. Then it was cleaned several times using deionized water as well as absolute ethanol and centrifuged to obtain a pure sample. Finally, samples were treated in a constant temperature drying oven at 80 °C for 8 h.

All experiments and tests involved chemicals are of analytical grade, and details are shown in Additional file 1.

### Microstructural Characterization

The crystal structures of as-prepared products are investigated by powder X-ray diffraction (XRD) measurements performed on a Rigaku D/max-3B instrument using Cu Kα radiation (40 kV, *λ* = 1.5406 Å, 2*θ* = 10°–80°). X-ray photoelectron spectroscopy (XPS, K-Alpha + , America) experiments were conducted in an ion-pumped chamber. The morphologies of the samples were characterized by field-emission scanning electron microscopy (FESEM, Quanta FEG 250, America) and transmission electron microscopy (TEM, Tecnai G2 F20 S-TWIN instrument with a field emission gun at 20 kV).

### Fabrication and Measurement of Gas Sensors

The fabrication progress of the gas sensors is as follows: First, the surfaces of the commercial Au interdigitated electrodes are cleaned with acetone, ethanol and deionized water. Then, 0.002 g of sample powder and 100 μL of deionized water are mixed and extracting 8–10 μL of sample mixed solution uniformly smeared onto a substrate of Au interdigitated electrodes. Finally, the coated electrode sheet is aged on a 230° hot bench for 24 h. Thus, the gas sensor is ready for testing.

**Fig. 1:**
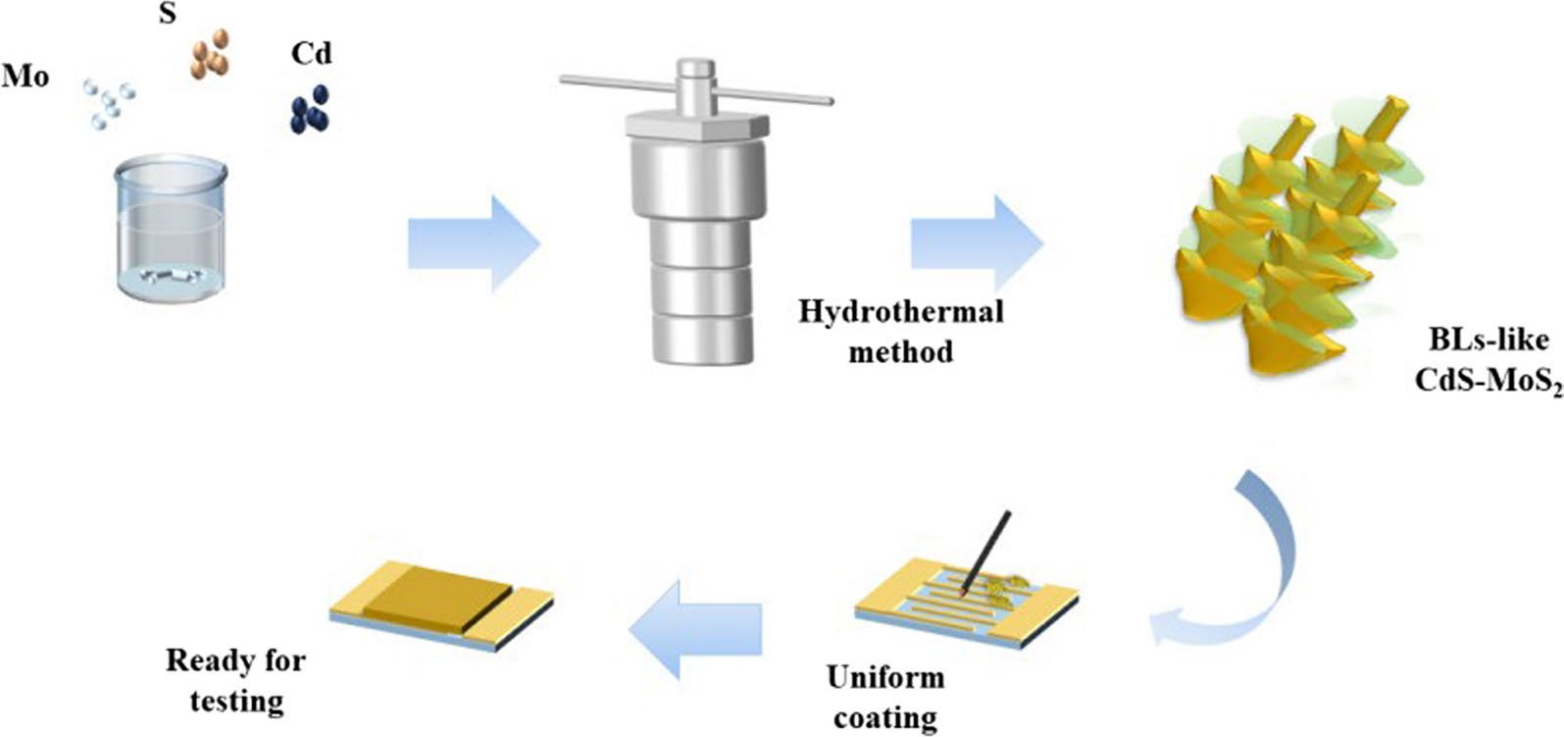
Diagram of CdS/MoS_2_ sensor

A full gas sensing platform (CGS-1TP, Beijing Elitetech Tech Co., Ltd. China) is utilized to estimate the gas sensing properties. The sample substrate forms a closed loop in close contact with the electrode of the platform (Additional file 1: Figure S1). Then, the system temperature rises to the set value and the sensor resistance decreases to a constant level. After that, the precalculated liquid is injected into the chamber (18 L). The resistance maintains stable and then open the chamber to restore the resistance of the sensor to the original level. All measurements are carried out in a well-ventilated laboratory, and the relative humidity is maintained at about 50% in the presence of a dehumidifier. The related preparation process is schematically shown in Fig. 1.

The response of the sensor at atmosphere or in target gas could be measured, which is defined as

1 $$S = \frac{{R_{{\text{a}}} }}{{R_{{\text{g}}} }}$$

where *R*_a_ is the resistance of the sensor at atmosphere and *R*_g_ is the resistance of sensor in the target gas.

## Results and Discussion

### Crystal Structure and Morphology

The X-ray diffractograms (XRD) analysis has investigated the crystal structures and phase composition of the samples (Additional file 1: Figure S2). All the main diffraction peaks at 24.9°, 26.5°, 28.2°, 43.8°, 47.8°, and 51.9° can be indexed to the hexagonal wurtzite phase of the CdS, which is perfectly coincided with the literature from the standard card (JCPDS card No. 41-1049) [25, 26]. In addition, because of the low content of MoS_2_ in the composite, two main observable diffraction peaks at 14.37° and 58.33° can be assigned to the (002) and (110) lattice plane of hexagonal MoS_2_ (JCPDS card No. 37-1492) [27–32].

The composition of the obtained composites is further analyzed by energy-dispersive X-ray spectrum (EDS). The distribution profiles of Cd, Mo and S are confirmed from the point analysis, as shown in Additional file 1: Figure S3. The appearance of these elements is more confident to confirm the presence of CdS and MoS_2_ in the composites, in which the carbon and the oxygen elements come from the conductive gel of the substrate. The excess oxygen may come from MoO_3_ generated in the synthesis or partial oxidation of MoS_2_.

**Fig. 2:**
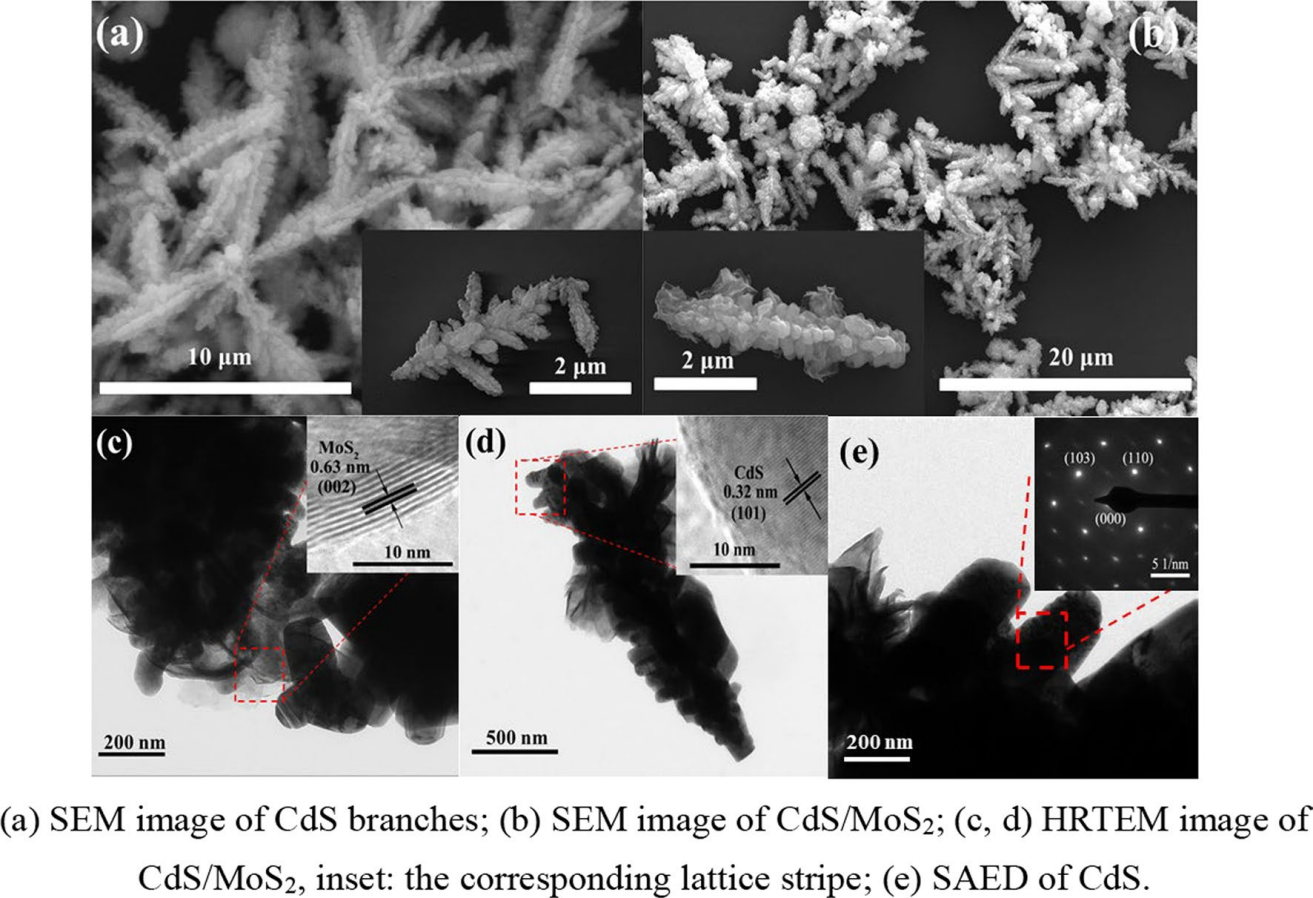
SEM, TEM and SAED images of CdS/MoS_2_ composites. (**a**) SEM image of CdS branches; (**b**) SEM image of CdS/MoS_2_; (**c**, **d**) HRTEM image of CdS/MoS_2_, inset: the corresponding lattice stripe; (**e**) SAED of CdS

The morphology and microstructure of the CdS branches and CdS/MoS_2_ composites are investigated by SEM and TEM experiments. Figure 2a, b demonstrates SEM images of the obtained CdS and CdS/MoS_2_, respectively. All samples exhibit similar morphologies. Compared with pure CdS branches, the layered structure is clearly attached to the front end of the branches. The morphology of the prepared samples is uniform, and the samples are randomly distributed on the substrate with a width of 2 μm and length up to 5 μm.

TEM and high-resolution TEM (HRTEM) images reveal that the CdS/MoS_2_ BLs consist of rod-like subunits. This close contact structure between CdS and MoS_2_ is beneficial for effectively electron transfer. Figure 2c shows the clear lattice fringes of the (002) planes of MoS_2_ with a d-spacing of 0.63 nm. In Fig. 2d, the lattice spacing of 0.32 nm is assigned to the (101) crystal planes of CdS. The structure of CdS covered with a few MoS_2_ layers facilitate gas sensing performance to boost, which originates from the favorable features of both CdS and MoS_2_ components. Selected area electron diffraction (SAED, Fig. 2e) reveals the single-crystalline properties of CdS, which is helpful for electron transport and block recombination reduction [24]. Based on the above-described evidences, it is confirmed that there is MoS_2_ in obtained CdS/MoS_2_ BLs.

**Fig. 3:**
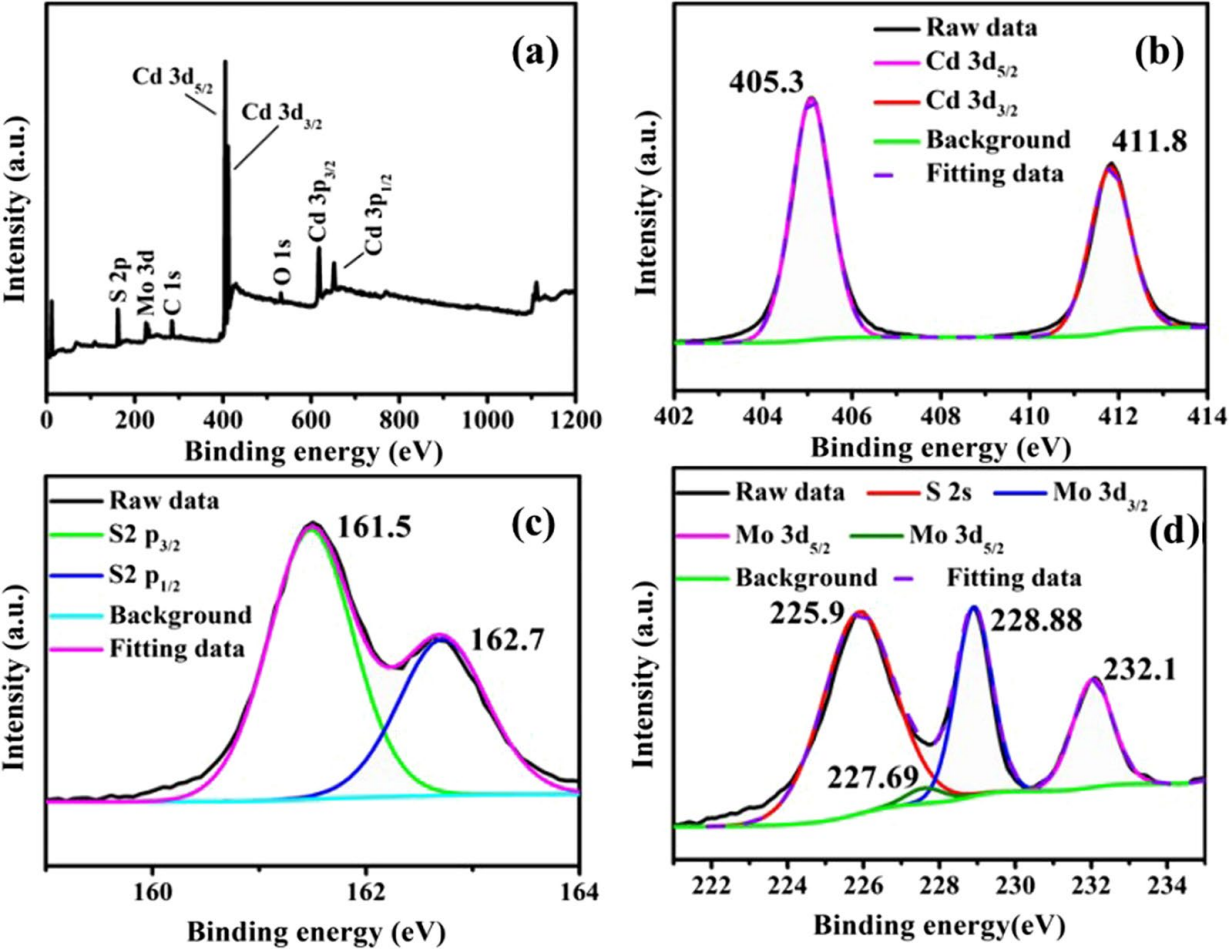
XPS spectra (**a**) full spectrum, (**b**) Cd 3*d*, (**c**) S 2*p*, (**d**) Mo 3*d* core level spectrum of CdS/MoS_2_ composites

X-ray photoelectron spectroscopy (XPS) is carried out to determine the valence electronic states of the atoms in the samples. The main spectrum indicated that the constituent elements are molybdenum, sulfur, cadmium and oxygen. Simultaneous carbon peaks (284.8 eV) are used to correct the calibration in Fig. 3a. As for oxygen peak, some oxygen has absorbed on the surface of the sample, or partial oxidation upon hydrothermal synthesis. In Fig. 3b, the peaks at 405.2 eV and 411.8 eV are assigned to the Cd^2+^ 3*d*_5/2_ and Cd^2+^ 3*d*_3/2_ regions, respectively [17]. In the S 2*p* spectrum (Fig. 3c), there are two double peaks belonging to MoS_2_ appeared at 161.5 eV (S^2−^ 2*p*_3/2_) and 162.7 eV (S^2−^ 2*p*_1/2_), respectively [33]. And XPS spectra of S 2*s* and Mo 3*d* are shown in Fig. 3d. The observed peak at 225.9 eV matches with the binding energy of S 2*s* in sulfides. In Mo 3*d* spectrum, two strong doublet peaks are detected, one is at 228.88 eV (Mo^4+^ 3*d*_3/2_) and the other is at 232.1 eV (Mo^4+^ 3*d*_5/2_), respectively [34]. In addition, a weak peak at 227.69 eV can be assigned to Mo 3d_5/2_ of Mo^4+^ in MoS_2_ [24]. Thus, it is reasonable to conclude that the MoS_2_ nanostructures should be successfully dispersed on CdS branches.

### VOCs Sensing Properties

**Fig. 4:**
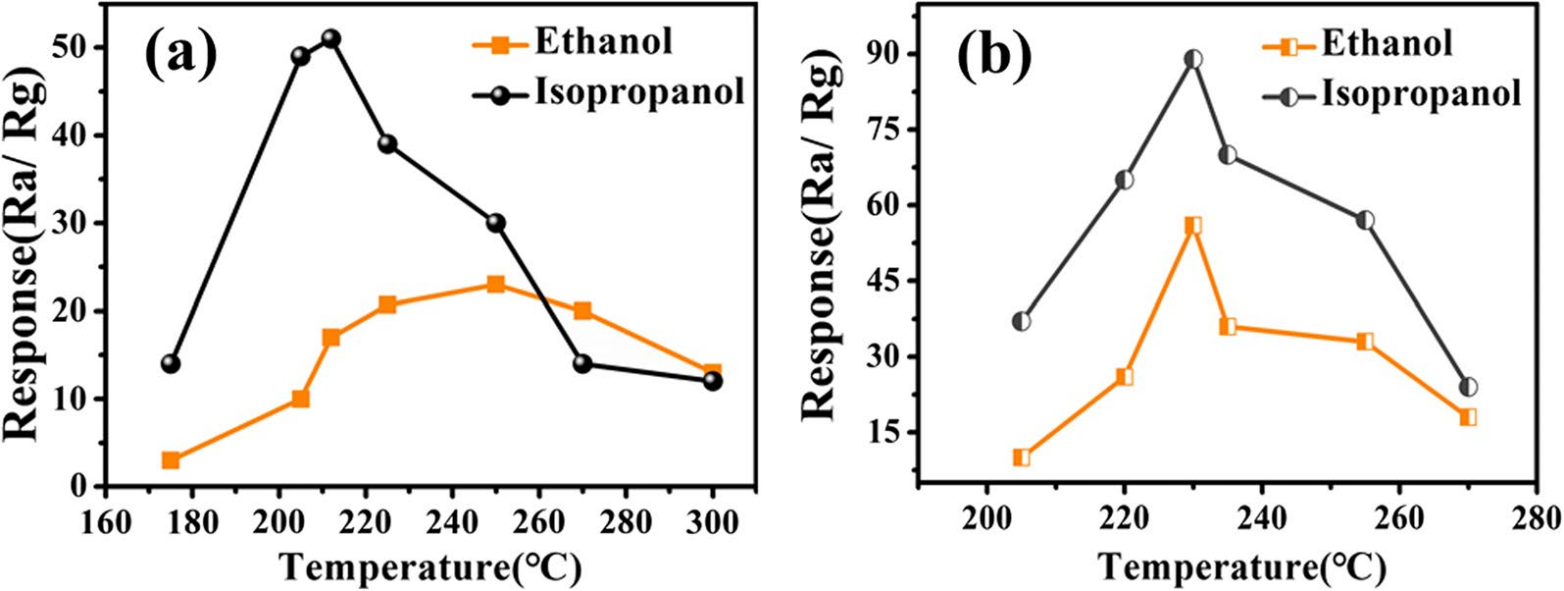
Response to ethanol and isopropanol (**a**) pure CdS (**b**) CdS/MoS_2_ composites

Sensing comparison of pure CdS branches and CdS/MoS_2_ BLs is shown in Fig. 4. It exhibits n-type semiconductors behavior with temperature dependence [35]. The obtained CdS/MoS_2_ BLs have stronger response to the same concentration than CdS branches. It may be due to the introduction of biomimetic structure that makes the carriers flow more efficiently. So the subsequent tests are based on the obtained CdS/MoS_2_ composites.

The operating temperature is an important parameter of gas sensing elements, which has a great influence on the surface states of the sensing materials. Figure 4b presents the gas-sensing responses versus temperature in the range of 200–270 °C for the sensor based on the CdS/MoS_2_ towards 100 ppm alcohols. It should be noted that the sensor has good response to ethanol and isopropanol. This observed phenomenon can be explained as follows: For most of the chemical resistance sensors, the interaction of gas molecules with the material surface is reflected by the change in the conductance of the material. At lower temperatures, there is no enough activation energy for the molecules to adsorb on the sensing channels of the material, thus leading to low response. In contrast, at higher temperatures desorption of molecules from the sensing channel accounts for the main effect rather than adsorption on the surface of it, which also leads to a lower response. In this work, the optimal response can be obtained at around 230 °C, at which *R*_a_ is 550 ΜΩ. The further measurements are carried out at 230 °C.

**Fig. 5:**
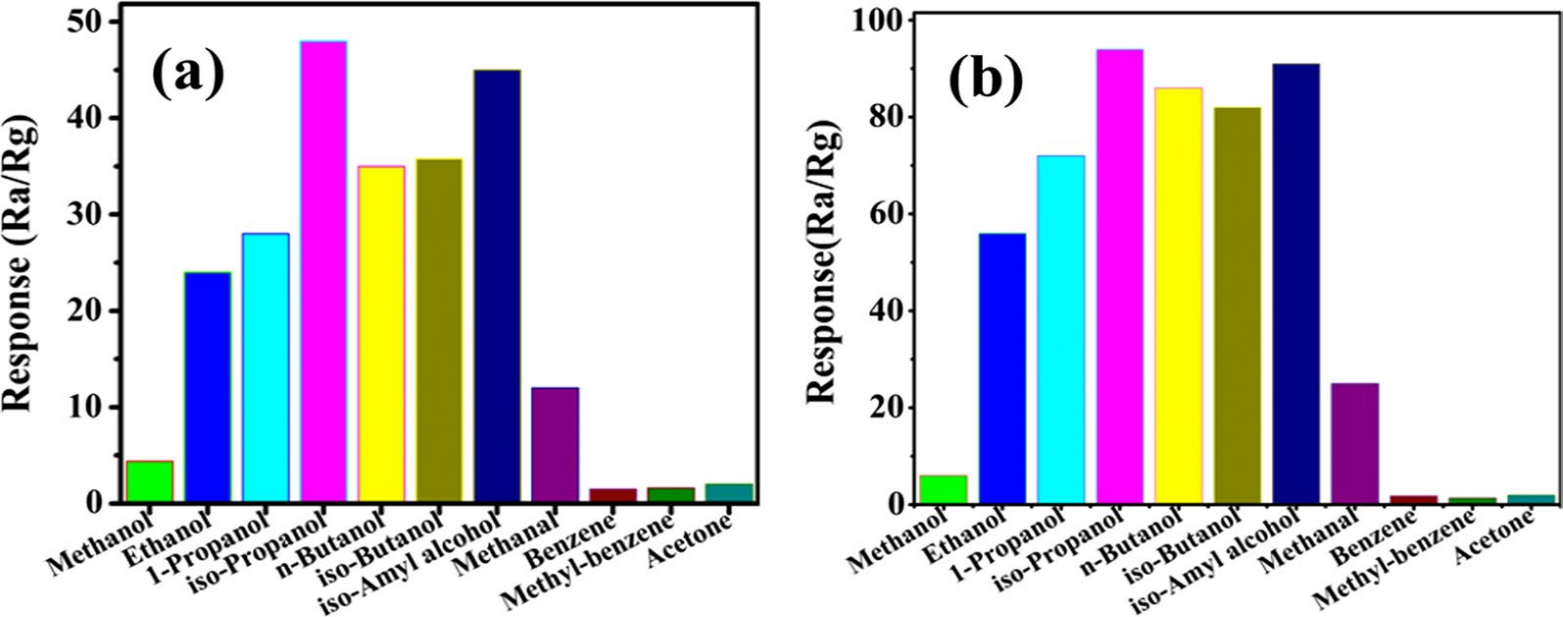
Comparison of (**a**) CdS branches and (**b**) CdS/MoS_2_ composites performance in detecting different VOCs under 100 ppm

Sensing responses of CdS branches (Fig. 5a) and CdS/MoS_2_ composites (Fig. 5b) for various VOCs under 100 ppm indicated that the proposed composites sensor displays an excellent selectivity for compounds containing OH groups in VOCs family. The response of composites sensor to VOCs is improved over twice higher than that of pure CdS branches. The possible mechanism is: the CdS and MoS_2_ favorably interact with the VOCs molecules containing OH groups, and the mobility of the carrier is increased after the reduction of MoS_2_. At the same time, it also revealed that the response to alcohols increased as increasing carbon atoms. This may be due to this fact that the alcohols with increment of (–CH_2_–)_*n*_ chain length are easily decomposed, and thus more molecules are absorbed and provide more electrons [36]. From the height of the histogram, it is intuitively seen that the composite sensor shows a unique high response to alcohols in VOCs.

**Fig. 6:**
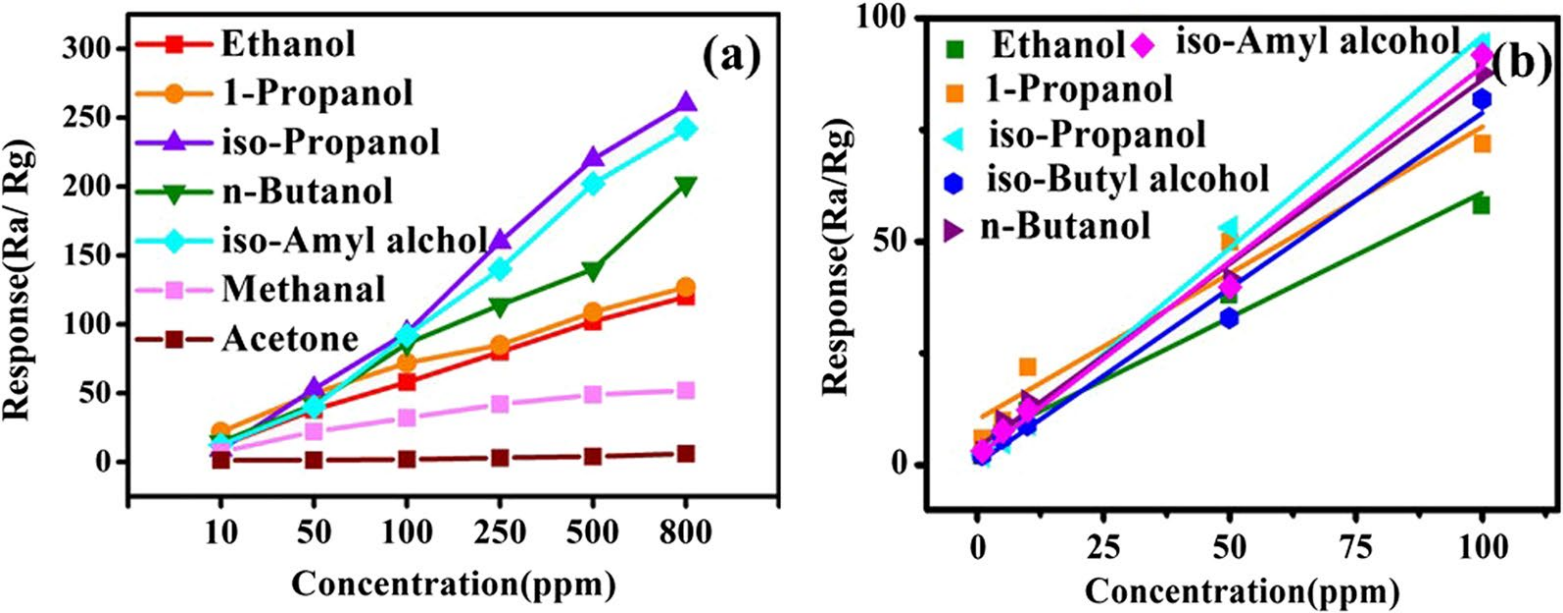
(**a**) The dynamic response of CdS/MoS_2_ composite to different gas. (**b**) The fitted lines of the response versus concentrations to six alcohols including ethanol, 1-propanol, isopropanol, n-butanol, iso-butyl alcohol, iso-amyl alcohol

To evaluate the performance of the CdS/MoS_2_ composites for alcohols, Fig. 6a shows the response curves versus concentration within the scope of 10–800 ppm to different VOCs. With increasing concentrations from 10 to 800 ppm, the upward linear relationships are displayed except for acetone and methanal. More importantly, it is found that the CdS/MoS_2_ composites exhibit a super-strong response to isopropyl alcohol.

Based on the previous results, the special selectivity of alcohols in VOCs is possibly related to the energy of the lowest unoccupied molecular orbital (LUMO). The lower the LUMO is, the stronger the electron trapping ability of gas molecules is [37, 38]. For example, the ethanol molecules in this work have lower LUMO than methanal ones. Therefore, the device has higher response to ethanol than methanal.

Figure 6b shows the fitting curves of response versus concentration of ethanol, 1-propanol, isopropanol, n-butanol, iso-butyl alcohol, and iso-amyl alcohol in the range of 1–100 ppm. It revealed an outstanding dependence of the response on concentration, suggesting that its response quickly increase at lower concentrations. The fitting equations and correlated coefficients (*R*^2^) are listed in Table 1. The closer the correlation coefficient is to the standard value of 1, the stronger the linear correlation between two variables and the better the fitting line of the equation.

**Table 1:** The comparison table of linear relationship fitting, *R*^2^ and LOD of different alcohols

Target gas	The linear relationship	*R*^2^	LOD/ppb
Ethanol	*Y* = 0.55836*X* + 4.86254	0.96213	84
1-Propanol	*Y* = 0.65553*X* + 10.2362	0.94699	152
Iso-propanol	*Y* = 0.94949*X* + 1.07708	0.9944	101
n-Butanol	*Y* = 0.8249*X* + 4.11331	0.99324	94
Iso-butyl alcohol	*Y* = 0.78424*X* + 0.42216	0.98243	81
Iso-amyl alcohol	*Y* = 0.87162*X* + 2.18232	0.98912	102

The theoretical limits of detection (LOD) for several alcohols are also calculated by the signal-to-noise ratio. Furthermore, the 40 consecutive points are selected to calculate the root mean square deviation (RMS). The LOD is expressed as [39, 40],

2 $$V_{2} = \sum \left( {y_{i} - y} \right)^{2}$$

3 $${\text{RMS}}_{{{\text{noise}}}} = \sqrt {\frac{{V_{2} }}{N}}$$

4 $${\text{LOD}}\left( {{\text{ppm}}} \right) = 3\frac{{{\text{rms}}}}{{{\text{slope}}}}$$

where *y*_*i*_ is the experimentally examined data, *y* is the corresponding calculated result by the fifth-order polynomial fitting of the measured data, and *N* is the number of selected data points. For instance, *V*_*χ2*_ and *N* of ethanol are 9.088 × 10^–3^ and 40, respectively. The noise of the sensor is 0.015, and the slope is 0.53985. Therefore, the LOD of the ethanol is 84 ppb, and implies that detect limit of the sensor can reach to ppb level. Our sensor has an excellent theoretical detection limit and applicable prospects for ethanol. For other gases, their corresponding parameters are also listed in Table 1.

**Fig. 7:**
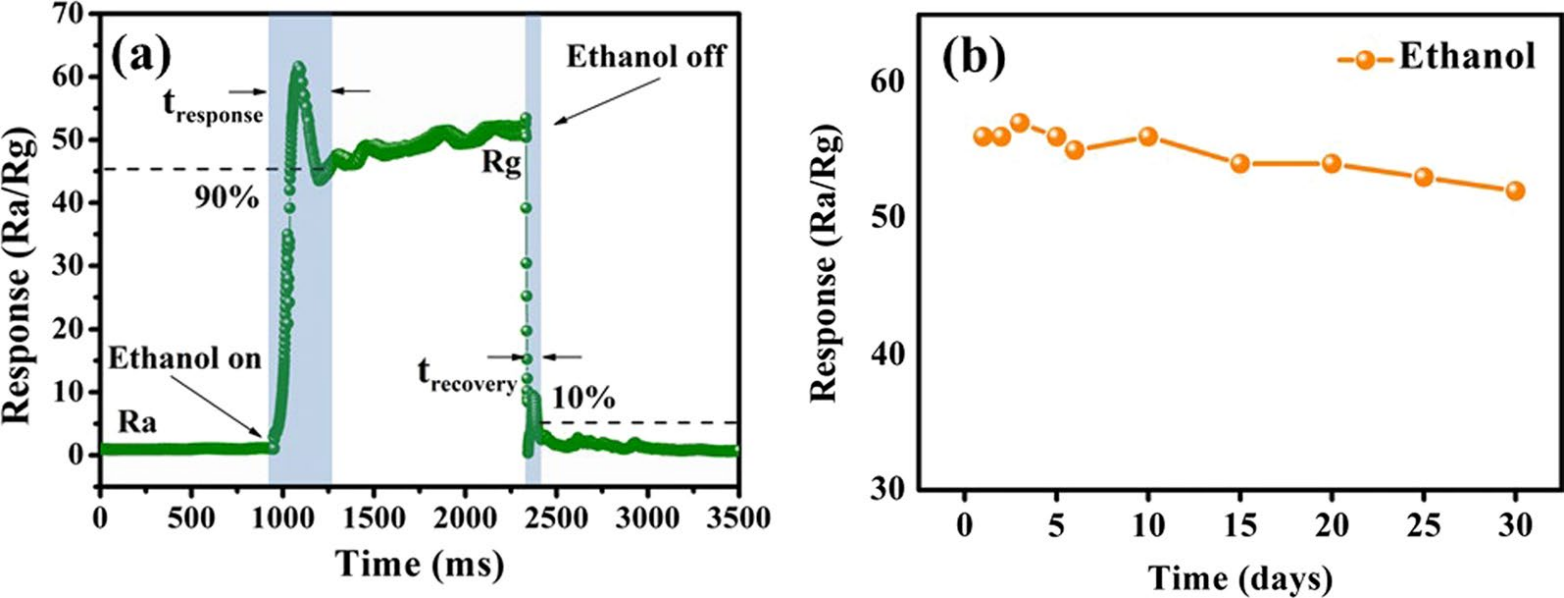
(**a**) Response and recovery time of CdS/MoS_2_ composites response to 100 ppm ethanol at working temperature. (**b**) The repeat measurement for 100 ppm ethanol within 30 days

Response and recovery time (*t*_res_ and *t*_rec_) of the device to 100 ppm ethanol at 230 °C are shown in Fig. 7a. The device resistance decreases from base resistance *R*_a_ = 550 MΩ in air to *R*_g_ = 10.5 MΩ as it exposed to 100 ppm of ethanol. It is calculated that *t*_res_ is approximately 32.5 s and *t*_rec_ is about 5.9 s. The examined vapor is not uniform at the initial stage, and the local high concentration is produced, which results in convexity in the rise curve.

The long-term stability of the sensor is assessed in 30 days, as indicated by the cyclicity in Fig. 7b. The response to 100 ppm ethanol slightly dropped from 56 to 53, indicating the high stability of the sensor. During this period, it is stored at atmosphere without special treatment.

**Fig. 8:**
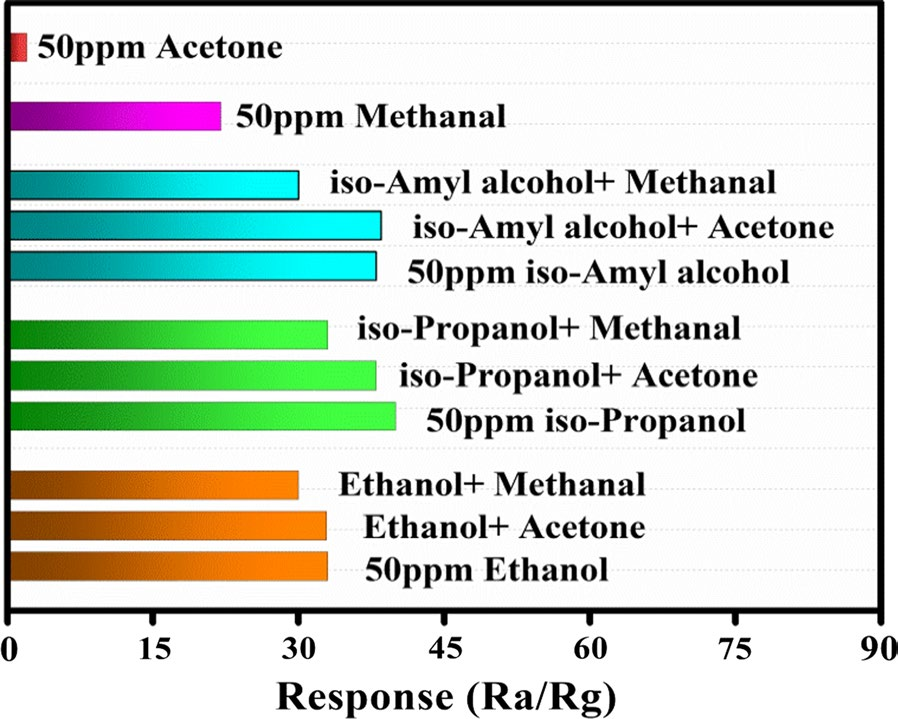
Response histogram of 50 ppm ethanol, isopropanol and isoamyl alcohol under the interference gas of 50 ppm acetone and 50 ppm methanal, respectively

In order to further investigate the anti-interference of the detector to VOCs, we study the response of 50 ppm alcohols (ethanol, isopropanol and isoamyl alcohol) under the interference of 50 ppm acetone and 50 ppm methanal, as shown in Fig. 8. It is seen that the relative deviations are − 9% (− 0.3%), − 17.5% (− 5%) and − 10.5% (0.013%), respectively for ethanol, isopropanol and isoamyl alcohol. Therefore, our sensor shows good anti-interference to methanol and acetone as it detected alcohols.

For comparison, the reported sensing parameters to ethanol and isopropanol are listed in Table 2. The proposed BLs CdS/MoS_2_ composites show excellent properties, indicating that our sensor has great potential for alcohols detection.

**Table 2:** Comparison of gas sensing performance of ethanol and isopropanol

Sensing materials	Target gas	Operating temperature (°C)	Concentration (ppm)	Response	References
NUM-CdS-3	Ethanol	230	100	20	[41]
CdS NWs	Ethanol	206	100	14.9	[16]
Leaf-like CdS	Iso-propanol	210	100	63	[42]
CdS nanoflakes	Iso-propanol	225	200	76	[17]
MoS_2_-SnO_2_	Ethanol	280	200	119	[23]
SnO_2_-ZnO	Ethanol	300	200	4.69	[43]
CuO/Cu_2_O	Ethanol	260	1000	27	[44]
Ag-ZnSnO_3_	Ethanol	200	100	83.96	[45]
Au-ZnSnO_3_	Ethanol	200	100	15.5	
CdS/MoS_2_ BLs	Ethanol	230	100	56	This work
	Iso-propanol		100	94	

## Mechanism

A possible mechanism is related to the surface control principle, which is conducive to gas adsorption, charge transfer, and desorption processes [46]. Oxygen at atmosphere can be adsorbed and attached to the surface of the sample, and subsequently maintain a homeostatic balance when the sensor is exposed to air. The following ionization reaction will occur with increasing temperature [41].

5 $${\text{O}}_{{2\left( {{\text{gas}}} \right)}} \to {\text{O}}_{{2\left( {{\text{ads}}} \right)}}$$

6 $${\text{O}}_{{2\left( {{\text{ads}}} \right)}} + {\text{e}}^{ - } \to 2{\text{O}}_{{2\left( {{\text{ads}}} \right)}}^{ - } , \, \left( {T < 100\;^\circ {\text{C}}} \right)$$

7 $${\text{O}}_{{2\left( {{\text{ads}}} \right)}} + {\text{e}}^{ - } \to 2{\text{O}}_{{\left( {{\text{ads}}} \right)}}^{ - } , \, \left( {100\,^\circ {\text{C}} < T < 300\,^\circ {\text{C}}} \right)$$

8 $${\text{O}}_{{\left( {{\text{ads}}} \right)}}^{ - } + {\text{e}}^{ - } \to {\text{O}}^{2 - } \left( {{\text{ads}}} \right), \, \left( {{\text{T }} > { 3}00\,^\circ {\text{C}}} \right)$$

**Fig. 9:**
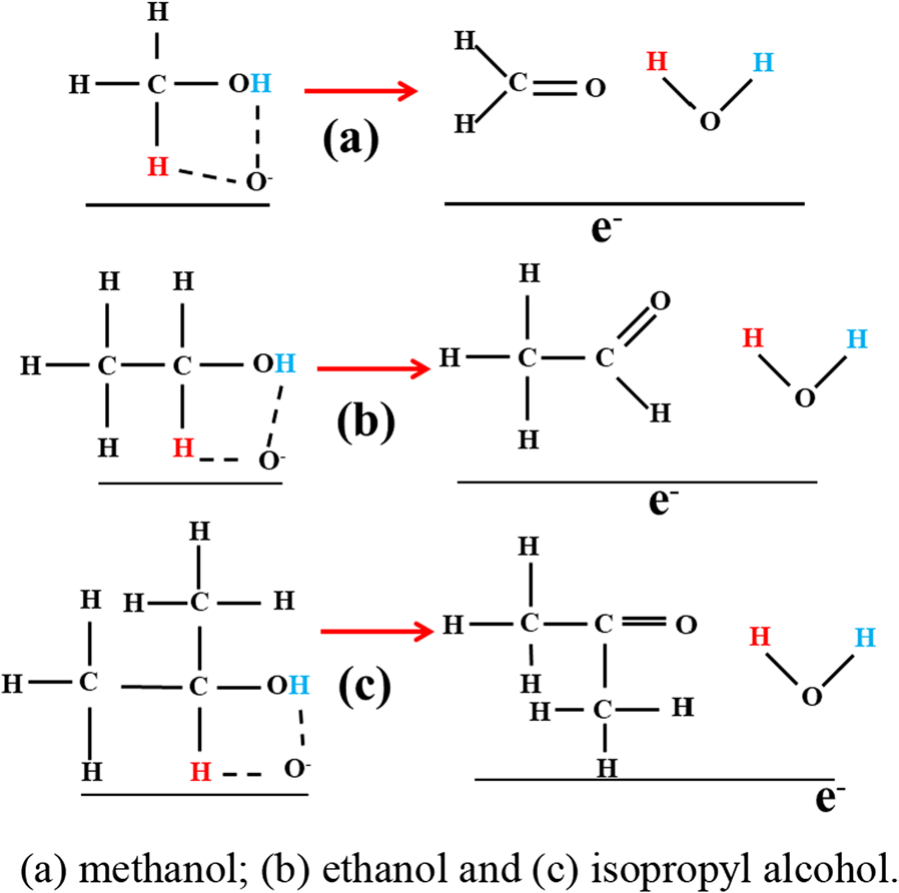
A possible alcohol adsorption mechanism. (**a**) Methanol; (**b**) ethanol and (**c**) isopropyl alcohol

The oxygen ions (O^−^) are believed to be dominant at the optimum operating temperature (230 °C). When methanol gas is injected into the chamber, Eq. (9) occurs and yields one H_2_O due to the attraction with O^−^_ads_, resulting in one electron into the material, as shown in Fig. 9a. Consequently, it causes the resistance to decrease to realize the detection of methanol. Thus, the dehydrogenated methanol turns into formaldehyde, and the reaction schematics is as follows:

9 $${\text{CH}}_{3} {\text{OH}} + {\text{O}}^{ - } \to {\text{CH}}_{2} {\text{O}} + {\text{H}}_{2} {\text{O}} + {\text{e}}^{ - }$$

A similar process will occur in the case of ethanol molecules and then turns into acetaldehyde. The reaction equation is denoted as

10 $${\text{CH}}_{3} {\text{CH}}_{2} {\text{OH}} + {\text{O}}^{ - } \to {\text{CH}}_{3} {\text{COH}} + {\text{H}}_{2} {\text{O}} + {\text{e}}^{ - }$$

Significant increase in response between methanol (CH_3_OH) and ethanol (CH_3_CH_2_OH) may be because of the different chemical structures of alcohols, and the dehydrogenation occurred at the CH_2_ group rather than the CH_3_ group, as illustrated in Fig. 9b. The reason is that compared CH_2_, the CH_3_ has higher electronegativity and attract more negative charges [36]. A similar situation occurs in isopropanol (Fig. 9c), dehydrogenation occurs in the CH group rather than the CH_2_ group. Therefore, isopropanol shows a high response, being different from the normal carbon chain law. For other alcohols, their response increases with increasing chain length of the alcohols, which is highly consistent with the phenomenon observed in the experiment.

## Conclusion

In this work, we prepared the CdS/MoS_2_ composites and then characterize their structure, morphology, composition and sensing properties. It is found that the sensor has excellent response to alcohols at working temperature, which increase as the chain length of alcohols becomes longer. The response values are 2.18/25/6/56/72/94/86/82/91 to 100 ppm of acetone/methanal/methanol/ethanol/1-propanol/isopropanol/n-butanol/iso-butanol alcohol/iso-amyl alcohol, respectively. The excellent performance on alcohols benefits from the branch and leaf shaped structure with a biomimetic framework. Moreover, theoretical LOD values are 84, 152, 101, 94, 81, 102 ppb for ethanol, 1-propanol, isopropanol, n-butanol, iso-butyl alcohol, iso-amyl alcohol, respectively. In addition, their superior anti-interference is obtained for alcohols detection in the mixture of methanal and acetone. Therefore, the BLs CdS/MoS_2_ composites are anticipated to be an outstanding potential candidate for detecting alcohols.

## Supplementary Information

**Additional file 1**. Details of materials required for additional experiments. Figure S1 The photograph of sensing test system and bench. Figure S2 XRD patterns of CdS/MoS2 composites. Figure S3 EDX point scan spectrum result.

## Data Availability

All data are fully available without restriction from the corresponding author on reasonable request.

## Abbreviations

VOCs: Volatile organic compounds
BLs: Branches and leaves
XRD: X-ray diffraction
XPS: X-ray photoelectron spectroscopy
FESEM: Field-emission scanning electron microscopy
TEM: Transmission electron microscopy
EDS: Energy-dispersive X-ray spectrum
LUMO: Lowest unoccupied molecular orbital
LOD: Limits of detection
