# Supplementary material for: Ultra-High Response Detection of Alcohols Based on CdS/MoS2 Composite
Source: Nanoscale Res Lett. 2022 Jan 6;17:7. doi: 10.1186/s11671-021-03647-3 (PMC8738843; doi:10.1186/s11671-021-03647-3)
Supplement: Supplementary file 1 — Additional file 1. Details of materials required for additional experiments. Figure S1 The photograph of sensing test system and bench. Figure S2 XRD patterns of CdS/MoS2 composites. Figure S3 EDX point scan spectrum result. [file 11671_2021_3647_MOESM1_ESM.docx]

**Supporting Information**

**Ultra-high response detection of alcohols based on CdS/MoS_2_ composite**

**Lei Liu^1,2^, Weiye Yang^1,2^, Hui Zhang^1,2^, Xueqian Yan^1,2^, Yingkai Liu^1,2,3,*^**

^1^Yunnan Key Laboratory of Opto-electronic Information Technology, Yunnan Normal University, Kunming 650500, China;

^2^ Institute of Physics and Electronic Information, Yunnan Normal University, Kunming 650500, China;

^3^ Key Laboratory of Advanced Technique & Preparation for Renewable Energy Materials, Ministry of Education, Yunnan Normal University, Kunming 650500, China;

^*^ Correspondence: ykliu@ynnu.edu.cn; Tel.: +86-871-6594-1166

**Materials**

Sodium molybdate (Na_2_MoO_4_·2H_2_O) is bought from Tianjin Chemical Reagent Factory No. 4 Kaida Chemical Plant (Tianjin, China). Cadmium nitrate tetrahydrate (Cd(NO_3_)_2_·4H_2_O) is obtained from Tianjin Damao Chemical Reagent Factory (Tianjin, China). Thiourea (CH_4_N_2_S), methanol, 1-propanol, isopropanol, methanal and xylene are purchased from Tianjin Fengchuan Chemical Reagent Technology Co., Ltd (Tianjin, China). Ethanol absolute and n-butanol are purchased from Tianjin Zhiyuan Chemical Reagent Co., Ltd. Acetone is obtained from Yunnan Yanglin Industrial Development Zone Shandian Pharmaceutical Co., Ltd. Iso-Amyl alcohol and iso-butyl alcohol are bought from Tianjin Jingdong Tianzheng Precision Chemical Reagent Factory.


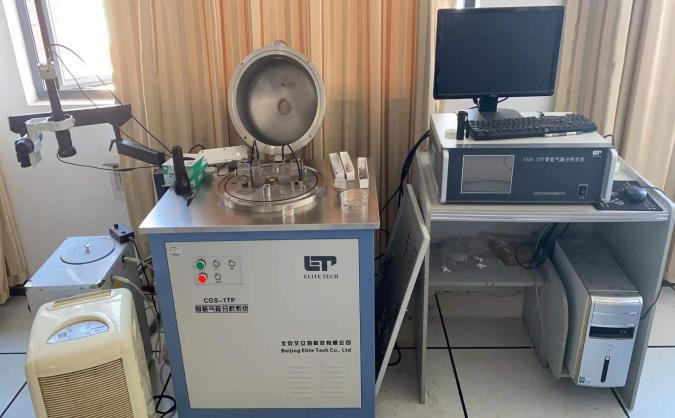

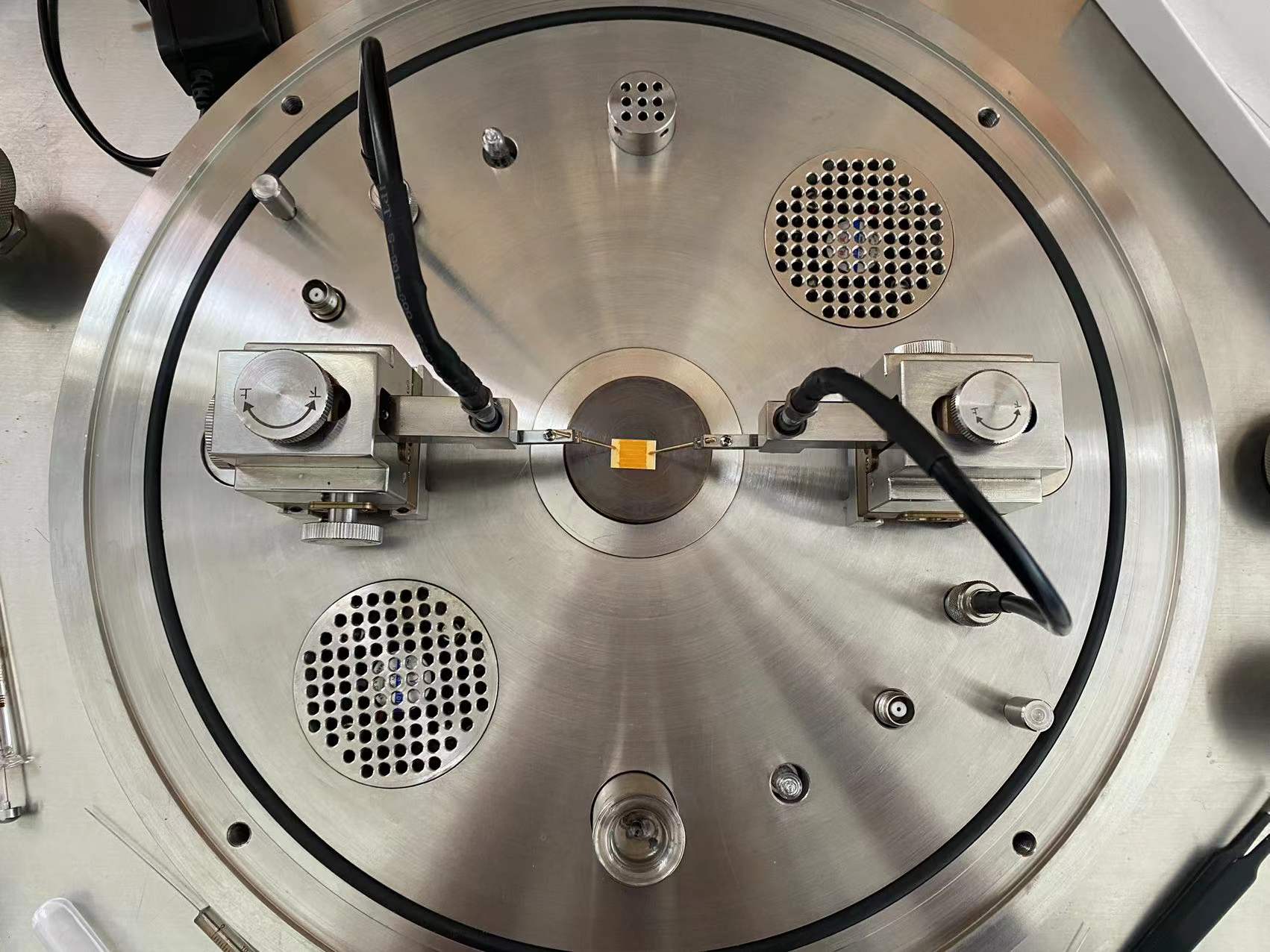


Figure S1 The photograph of sensing test system and bench.


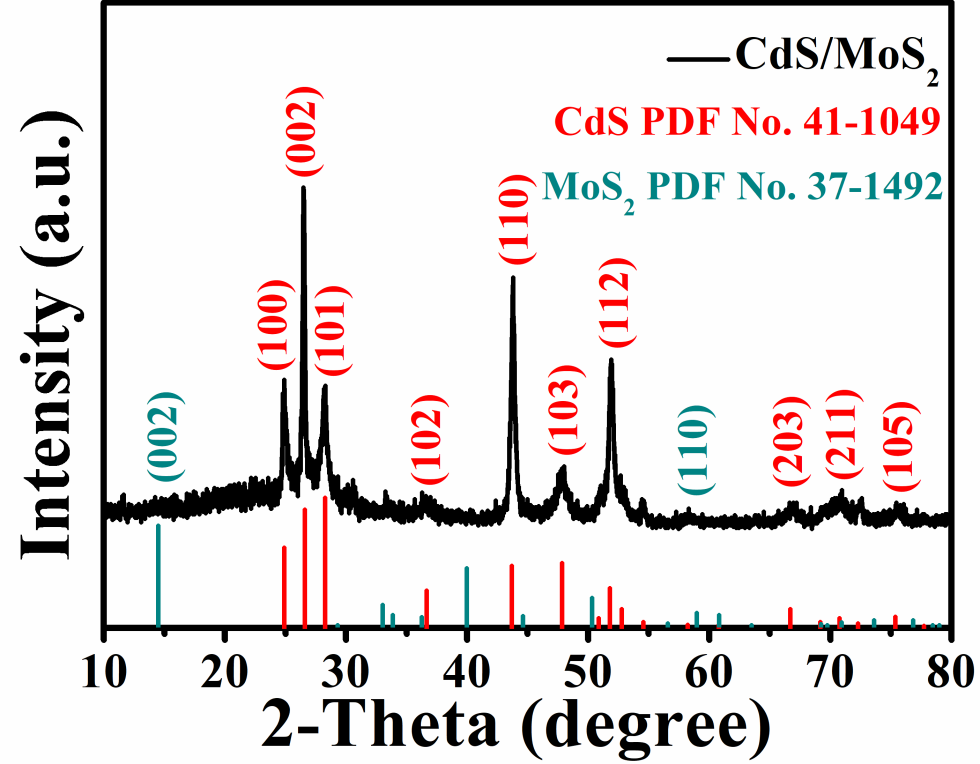


Figure S2 XRD patterns of CdS/MoS_2_ composites.


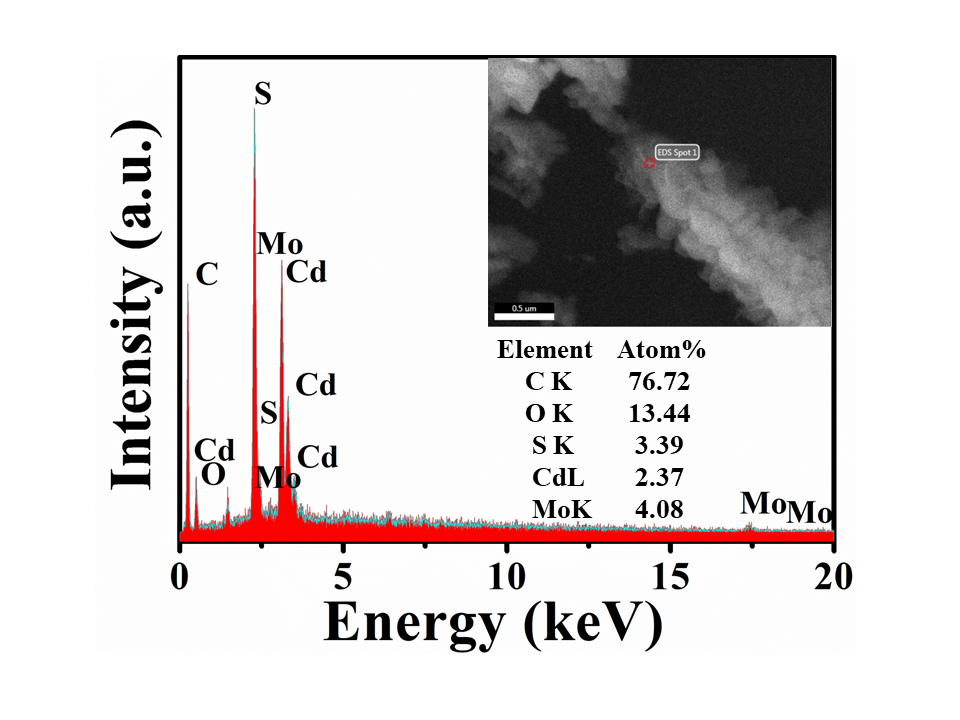


Figure S3 EDX point scan spectrum result and the element ratio.
